# Family-based factors associated with overweight and obesity among Pakistani primary school children

**DOI:** 10.1186/1471-2431-11-114

**Published:** 2011-12-16

**Authors:** Muhammad Umair Mushtaq, Sibgha Gull, Ubeera Shahid, Mahar Muhammad Shafique, Hussain Muhammad Abdullah, Mushtaq Ahmad Shad, Arif Mahmood Siddiqui

**Affiliations:** 1 Ubeera Memorial Research Society, Allama Iqbal Medical College, Lahore, 54000 Punjab, Pakistan; 2 District Health Office Nankana Sahib, Punjab Department of Health, Nankana Sahib, 39100 Punjab, Pakistan

## Abstract

**Background:**

Childhood obesity epidemic is now penetrating the developing countries including Pakistan, especially in the affluent urban population. There is no data on association of family-based factors with overweight and obesity among school-aged children in Pakistan. The study aimed to explore the family-based factors associated with overweight and obesity among Pakistani primary school children.

**Methods:**

A population-based cross-sectional study was conducted with a representative multistage cluster sample of 1860 children aged five to twelve years in Lahore, Pakistan. Overweight (> +1SD BMI-for-age z-score) and obesity (> +2SD BMI-for-age z-score) were defined using the World Health Organization reference 2007. Chi-square test was used as the test of trend. Linear regression was used to examine the predictive power of independent variables in relation to BMI. Logistic regression was used to quantify the independent predictors of overweight and adjusted odds ratios (aOR) with 95% confidence intervals (CI) were obtained. All regression analyses were controlled for age and gender and statistical significance was considered at P < 0.05.

**Results:**

Significant family-based correlates of overweight and obesity included higher parental education (P < 0.001), both parents working (P = 0.002), fewer siblings (P < 0.001), fewer persons in child's living room (P < 0.001) and residence in high-income neighborhoods (P < 0.001). Smoking in living place was not associated with overweight and obesity. Higher parental education (P < 0.001) and living in high-income neighborhoods (P < 0.001) showed a significant independent positive association with BMI while greater number of siblings (P = 0.001) and persons in child's living room (P = 0.022) showed a significant independent inverse association. College-level or higher parental education as compared to high school-level or lower parental education (aOR 2.54, 95% CI 1.76-3.67), living in high-income neighborhoods as compared to low-income neighborhoods (aOR 2.13, 95% CI 1.31-3.46) and three or less siblings as compared to more than three siblings (aOR 1.75, 95% CI 1.26-2.42) were significant independent predictors of overweight.

**Conclusion:**

Family-based factors were significantly associated with overweight and obesity among school-aged children in Pakistan. Higher parental education, living in high-income neighborhoods and fewer siblings were independent predictors of overweight. These findings support the need to design evidence-based child health policy and implement targeted interventions, considering the impact of family-based factors and involving communities.

## Background

Obesity is a global epidemic and children are the worst affected with an estimated ten percent of school-aged children being overweight and one quarter of these being obese worldwide [1,2]. The 2004 World Health Assembly at Geneva called for specific action to halt the epidemic that is now penetrating the developing countries including Pakistan, especially in the affluent urban population [3-5]. Childhood obesity adversely affects physiological and psychosocial well-being, results in cardiovascular and metabolic diseases, leads to increased mortality and morbidity, and causes heavy health expenditures and reduced social status [1,6-8]. Targeted interventions for the prevention of childhood obesity, tailored to local circumstances and involving communities, should begin early in life [9].

Influence of family environment and parental characteristics has important consequences regarding childhood obesity and it should be considered in designing policies and interventions [10-12]. Globally, the relationship between childhood obesity and family-based factors, including parental education, parental working status, siblings, persons in child's living room, smoking in living space and neighborhood income level has been extensively explored [13-32]. However, most studies are conducted in the developed countries and literature is scarce in this regard among South Asian children. There is no data on association of family-based factors with overweight and obesity among school-aged children in Pakistan. The study aimed to explore family-based factors associated with overweight and obesity among Pakistani primary school children.

## Methods

### Design, setting and sample

A population-based cross-sectional study titled 'Nutritional Assessment among School-going Children in Lahore, Pakistan (NASCL)' was conducted among primary school children aged five to twelve years in 2009-2010. Lahore, a metropolis with multiethnic populations, is the capital of Pakistan's most populous province Punjab. It has a population of nine million including 2.5 million primary school children, and 81% of the population resides in the urban area (Administrative data, Government of the Punjab, 2010).

A stratified, multistage cluster sample of 1860 children aged five to twelve years in twelve primary schools of City District Lahore was enrolled. The sampling design has been used previously in nutritional assessment surveys [19,28,29,33-35]. Stratified sampling, based on the population and educational system characteristics, was used to have proportionate representation of gender, area of residence and socioeconomic status (SES). The list of all public and private primary schools in Lahore was provided by the Punjab Department of Education. The listed schools were stratified according to the geographic area and monthly fee structure of schools into following four strata: a) urban with high SES (urban area and fee > 2500 PKR), b) urban with middle SES (urban area and fee = 1000-2500 PKR), c) urban with low SES (urban area and fee < 1000 PKR), and d) rural with low/disadvantaged SES (rural area and fee ~100 PKR or free). The former two strata included private (including public-private mix) schools and the later two strata included public schools. In Pakistan, public schools cater low SES urban and rural children while high SES and middle SES urban children are educated in private and public-private mix schools. Three schools were selected at random from each stratum and contacted by the Departments of Education and Health to participate voluntarily in the study. If the school administration refused to participate, next school was selected randomly from the respective stratum. For each school, a list of all classes in five grades (one to five) was obtained and one class in each grade was selected at random. In this way, sixty classes, five from each school, were selected. For each of the selected classes, first thirty-one children on class attendance register, present on data collection day and aged five to twelve years, were included in the study. Participation of children in the study was voluntary. Children suffering from any known metabolic syndrome (e.g. Prader-Willi syndrome) were excluded. Sample size was calculated using Epi Info 6.04d (United States Centers for Disease Control and Prevention, 2004) with a confidence (1-α) of 95%, anticipated prevalence of 5% and margin of error of ± 1. The minimum sample size calculated was 1823 and a sample of 1860 was deemed sufficient.

### Data Collection

The sampled schools were visited on pre-arranged dates in summer 2009 by a team of trained senior medical students lead by the Principal Investigator. Health education of children and teachers was also carried out after data collection in the respective school. Analogue physician health scales, standardized before the examination, were used [36]. Height in centimeters (cm) and weight in kilograms (kg) were measured following the standard procedure to the nearest 0.1 cm and 0.5 kg respectively. The child was asked to stand relax, feet were placed together with heels, buttocks and shoulder blades against the stick and head was positioned in the Frankfurt horizontal plane. All measurements were taken in light summer school uniform without shoes during mornings or early afternoons. Most frequently used measure for obesity is body mass index (BMI), defined as weight (kg)/height squared (m^2^), and BMI-for-age is the anthropometric index of relative weight recommended by the international expert committees [37].

For each of the sampled classes, demographic information of all officially enrolled students was obtained before data collection, including gender, date of birth, residential address and parental education. Demographic information of students not found on official rosters but currently enrolled in that class was obtained from class teachers. Among measures of social class, parental education had been most strongly related to childhood obesity [14,15]. Parental education level was based on the parent with the highest total years of schooling and neighborhood income level was based on the approximate income estimate of child's residential area obtained from the Revenue Department of City District Government Lahore.

The study instrument was a structured questionnaire, designed in English. Study instruments and procedures were pre-tested in the field and modified accordingly. The questionnaire included questions regarding family-based factors including parental working status, siblings, persons in child's living room and smoking in living space [38,39]. Senior medical students trained in the interviewing techniques interviewed children in presence of their class teacher (guardian). Each child was asked regarding whether his/her mother works outside or is she a housewife, how many older/younger siblings he/she has and how many persons are living in his/her living room? Smoking was defined as smoking tobacco by cigarette, cigar, pipe or hookah. Hookah (water pipe) is a single or multi-stemmed instrument used for smoking tobacco in South Asia.

Quality control measures and good practices including training, pre-testing the processes and materials, field monitoring of data collection, logistics management and daily meetings of the study teams were ensured. Informed consent statement was printed on the form. Verbal informed consent for the child to participate in the study was taken from class teachers and school heads. As the study involved no invasive procedure, verbal informed consent was deemed sufficient. The study was approved by the Ethical Review Board of Allama Iqbal Medical College, Lahore. Permissions to conduct the study were granted by the Punjab Departments of Education and Health, and the sampled schools.

### Statistical Analysis

Data were entered and analyzed by manual and computerized checking using SPSS version 18.0 (SPSS Inc. Chicago IL, United States, 2009). Age was calculated to the precise day by subtracting the date of birth from the date of examination. The z-score values for BMI-for-age were calculated using the World Health Organization's software, AnthroPlus, for assessing growth of the world's children and adolescents (WHO, 2009). Overweight (> +1SD BMI-for-age z-score) and obesity (> +2SD BMI-for-age z-score) were defined using the WHO reference 2007 [40,41].

Bivariate analysis, using chi-square test as the test of trend, was conducted to compare differences in the prevalence of overweight and obesity relative to family-based factors. Crude odds ratios (OR) with 95% confidence interval (CI) were calculated to examine the relationship between overweight and family-based factors by univariate analyses. Linear regression was used to examine the predictive power of family-based factors (independent variables) in relation to BMI (dependent variable). Multivariate logistic regression was used to estimate the simultaneous effect of several covariates on a dichotomous outcome. Parental education, parental working status, siblings, persons in child's living room and neighborhood income were entered into the multivariate model concurrently to quantify their independent importance for risk of being overweight and adjusted odds ratios (aOR) with 95% CI were obtained. All regression analyses were controlled for age and gender. Statistical significance was considered at P < 0.05 and all tests were two-sided.

## Results

The study included a sample of 1860 primary school children aged five to twelve years. The male-female ratio was 1.11 with 52.5% boys and 47.5% girls. The sample involved 20% children from each grade and 25% children from each area and SES stratum. Twenty percent parents were illiterate followed by those educated up to high school (27%), college (28%) and higher (25%). Majority of children had one to three siblings (54%) followed by more than three siblings (44%) and no sibling (1%). Most children (51%) had more than three persons in living room followed by one to three persons (43%) and no person (6%). Smoking in living place was 30%. Most children (49%) lived in middle-income neighborhoods followed by low-income (35%) and high-income (16%) neighborhoods. Median age (range) was 8(5-12) years and mean age (SD) was 8.49 (1.81) years. Mean (SD) BMI was 20.7 (5.02) kg/m^2 ^and mean (SD) BMI-for-age z score was -0.3 (1.5). Fifty-one percent children had normal weight status (-1SD to +1SD BMI-for-age z-score), 9.5% children were overweight (> +1SD to +2SD BMI-for-age z-score) and 7.5% children were obese (> +2SD BMI-for-age z-score) while under-nutrition or thinness (< -1SD BMI-for-age z-score) was observed in 32% children.

**Figure 1:**
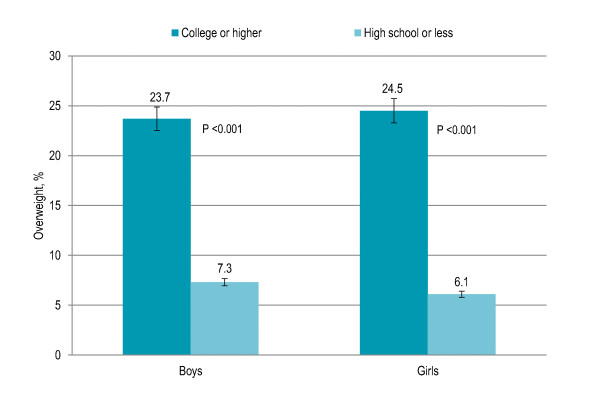
**Gender-specific prevalence (with confidence interval bars) of overweight among Pakistani primary school boys (n = 977) and girls (n = 883) by parental education level**.

**Figure 2:**
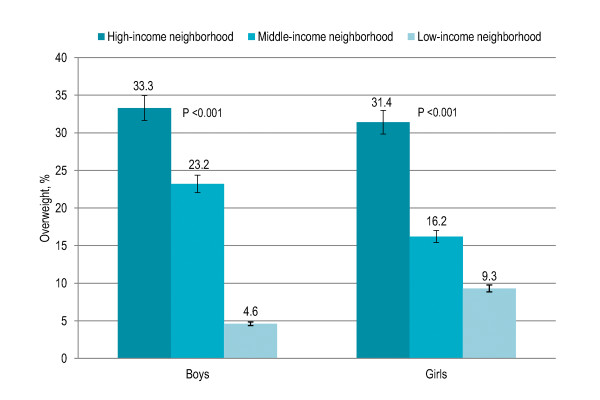
**Gender-specific prevalence (with confidence interval bars) of overweight among Pakistani primary school boys (n = 977) and girls (n = 883) by neighborhood income level**.

Association of family-based factors with overweight and obesity is shown in Table 1. Children whose parents were having college (23%) or higher (29%) education had significantly higher risk of being overweight and obese (P < 0.001) as compared to children whose parents were illiterate (3%) and educated up to high school (10%). Overweight and obesity were significantly higher among children with parents having higher education among both boys and girls (both P < 0.001) [Figure 1]. Children whose both parents were working (22.5%) were significantly more likely to be overweight and obese (P = 0.002) than those whose mother was a housewife (15.5%). Overweight and obesity were 9% among children having more than three siblings that significantly increased to 23% among children having one to three siblings and 35% among children having no sibling (P < 0.001). Twelve percent children having more than three persons in living room were overweight and obese that significantly increased to 20% among children with one to three persons in living room and 38% among children with no person in living room (P < 0.001). Smoking in living place was not associated with overweight and obesity. Children living in high-income neighborhoods (32%) were significantly more likely to be overweight and obese (P < 0.001) than those living in middle-income (19.5%) and low-income (6.5%) neighborhoods. Higher overweight and obesity prevalence in high-income neighborhoods was observed among both boys and girls (both P < 0.001) [Figure 2].

**Table 1:** Association of family-based factors with overweight and obesity among Pakistani primary school children (n = 1860)

	Total (n = 1860)	Thin^a^ (n = 601)	Normal^b^ (n = 943)	Overweight^c^ (n = 176)	Obese^d^ (n = 140)	Significance	
				
Family-based factors	n (%)	n (%)	n (%)	n (%)	n (%)	χ^2^	P value
**Parental education**							
Illiterate	366 (19.7)	163 (44.5)	193 (52.7)	8 (2.2)	02 (0.5)	161.56	< 0.001
High school	496 (26.7)	193 (38.9)	255 (51.4)	33 (6.7)	15 (3.0)		
College	531 (28.5)	142 (26.7)	268 (50.5)	66 (12.4)	55 (10.4)		
Higher education	467 (25.1)	103 (22.1)	227 (48.6)	69 (14.8)	68 (14.6)		
**Parental working status**							
Father only	1465 (78.8)	496 (33.9)	742 (50.6)	125 (8.5)	102 (7.0)	14.32	0.002
Both parents	395 (21.2)	105 (26.6)	201 (50.9)	51 (12.9)	38 (9.6)		
**Siblings**							
No	26 (1.4)	6 (23.1)	11 (42.3)	4 (15.4)	5 (19.2)	98.33	< 0.001
1-3	1008 (54.2)	262 (26.0)	511 (50.7)	121 (12.0)	114 (11.3)		
> 3	826 (44.4)	333 (40.3)	421 (51.0)	51 (6.2)	21 (2.5)		
**Persons in child's living room**							
No	116 (6.2)	24 (20.7)	48 (41.4)	28 (24.1)	16 (13.8)	77.84	< 0.001
1-3	791 (42.5)	212 (26.8)	420 (53.1)	86 (10.9)	73 (9.2)		
> 3	953 (51.2)	365 (38.3)	475 (49.8)	62 (6.5)	51 (5.4)		
**Smoking in living place**							
Yes	546 (29.4)	194 (35.5)	263 (48.2)	52 (9.5)	37 (6.8)	4.04	0.731
No	1314 (70.6)	407 (31.0)	680 (51.8)	124 (9.4)	103 (7.8)		
**Neighborhood income**							
Low	651 (35.0)	293 (45.0)	316 (48.5)	28 (4.3)	14 (2.2)	145.68	< 0.001
Middle	910 (48.9)	247 (27.1)	486 (53.4)	98 (10.8)	79 (8.7)		
High	299 (16.1)	61 (20.4)	141 (47.2)	50 (16.7)	47 (15.7)		

^a ^< -1SD BMI-for-age z-score relative to the World Health Organization reference 2007

^b^-1SD to +1SD BMI-for-age z-score relative to the World Health Organization reference 2007

^c ^> +1SD to +2SD BMI-for-age z-score relative to the World Health Organization reference 2007

^d ^> +2SD BMI-for-age z-score relative to the World Health Organization reference 2007

College-level or higher parental education as compared to high school-level or lower parental education (aOR 2.54, 95% CI 1.76-3.67), living in high-income neighborhoods as compared to low-income neighborhoods (aOR 2.13, 95% CI 1.31-3.46) and three or less siblings as compared to more than three siblings (aOR 1.75, 95% CI 1.26-2.42) were significant independent predictors of overweight [Table 2]. Higher parental education (P < 0.001) and living in high-income neighborhoods (P < 0.001) showed a significant independent positive association with BMI while greater number of siblings (P = 0.001) and persons in child's living room (P = 0.022) showed a significant independent inverse association with BMI [Table 3].

**Table 2:** Logistic regression analysis of family-based factors associated with overweight among Pakistani primary school children (n = 1259)

	Total^a^ (n = 1259)	Normal weight^b ^(n = 943)	Overweight^c ^(n = 316)	Crude OR (95% CI)	P Value	Adjusted OR (95% CI)^d^	P Value
Family-based factors	n (%)	n (%)	n (%)				
**Parental education**							
High school or less	506 (40.2)	448 (88.5)	58 (11.5)	Reference	-	Reference	-
College or higher	753 (59.8)	495 (65.7)	258 (34.3)	4.03 (2.95-5.50)	< 0.001	2.54 (1.76-3.67)	< 0.001
**Parental working status**							
Father only	969 (77.0)	742 (76.6)	227 (23.4)	Reference	-	Reference	-
Both parents	290 (23.0)	201 (69.3)	89 (30.7)	1.45 (1.08-1.94)	0.013	0.85 (0.62-1.16)	0.306
**Siblings**							
≤3	766 (60.8)	522 (68.1)	244 (31.9)	2.73 (2.04-3.66)	< 0.001	1.75 (1.26-2.42)	0.001
> 3	493 (39.2)	421 (85.4)	72 (14.6)	Reference	-	Reference	-
**Persons in child's living room**							
≤3	671 (53.3)	468 (69.7)	203 (30.3)	1.82 (1.40-2.37)	< 0.001	1.11 (0.83-1.49)	0.480
> 3	588 (46.7)	475 (80.8)	113 (19.2)	Reference	-	Reference	-
**Neighborhood income**							
Low	358 (28.4)	316 (88.3)	42 (11.7)	Reference	-	Reference	-
Middle	663 (52.7)	486 (73.3)	177 (26.7)	2.74 (1.90-3.95)	< 0.001	1.39 (0.91-2.13)	0.129
High	238 (18.9)	141 (59.2)	97 (40.8)	5.18 (3.43-7.82)	< 0.001	2.13 (1.31-3.46)	0.002

^a^The model excludes under-nourished children having a BMI-for-age z-score of < -1SD relative to the World Health Organization reference 2007 (n = 601)

^b^-1SD to +1SD BMI-for-age z-score relative to the World Health Organization reference 2007

^c ^> +1SD BMI-for-age z-score relative to the World Health Organization reference 2007 (includes obese children)

^d^The model is adjusted for age and gender

**Table 3:** Linear regression analysis of family-based factors with BMI among Pakistani primary school children (n = 1860)^a,b^

Characteristics	Regression coefficient (95% CI)	Standard error	P value
Higher parental education	0.68 (0.46 to 0.90)	0.11	< 0.001
Both parents working (employed)	0.31 (-0.16 to 0.77)	0.24	0.201
Greater number of siblings	-0.70 (-1.11 to -0.28)	0.21	0.001
Greater number of persons in child's living room	-0.40 (-0.73 to -0.06)	0.17	0.022
Higher neighborhood income	1.07 (0.73 to 1.40)	0.17	< 0.001

^a^The model is adjusted for age and gender.

^b^R^2 ^for the model = 0.329.

## Discussion

This was the first study with a representative sample to explore association of family-based factors with overweight and obesity among Pakistani school-aged children. Higher parental education was significantly associated with overweight and obesity among both boys and girls. Children with parents having college-level or higher education were independently more likely to be overweight as compared to children with parents having high school-level or lower education. Higher parental education was independent predictor of higher BMI. Positive association of childhood obesity with higher parental education had been observed in the developing countries [16,21]; however, studies in the developed countries had shown inverse association of parental education with obesity [18,19,42]. Children whose both parents were working had significantly higher rates of overweight and obesity than those whose mother was a housewife; however, in adjusted regression analyses, the effect did not remain significant. Maternal employment had been associated with childhood obesity previously [20]. Female employment increases family income contributing to improved child health; however, it often adversely affects child nutrition because of the effects on breastfeeding and maternal care-giving [43].

Fewer siblings and less crowded housing are indirect indicators of higher socioeconomic class [38,39]. Fewer siblings and fewer persons in child's living room had significant association with overweight and obesity, and both factors showed a significant independent inverse association with BMI. These findings are consistent with previous studies [19,21,22]. Children having three or less siblings were independently more likely to be overweight as compared to children having more than three siblings. Overweight was not significantly associated with persons in child's living room in regression analysis adjusted for all family-based factors. Smoking in living place had been associated with overweight and obesity in previous literature [17,19,20,22,23], but no association was observed in the present study.

Children living in high-income neighborhoods were more likely to be overweight and obese as compared to children living in middle-income and low- income neighborhoods, and the trend was significant among both boys and girls. Higher neighborhood income showed a significant independent association with higher BMI. Children living in high-income neighborhoods were independently more likely to be overweight as compared to children living in low-income neighborhoods. Childhood obesity has been associated with higher socioeconomic class in the developing countries [24-27,42,44]; however, studies in the developed world have shown inverse association of socioeconomic class with childhood obesity [15,28-31,45-47]. Different socio-cultural circumstances in the developing countries undergoing nutrition transition explain the contradiction and association between socioeconomic factors and over-nutrition vary in societies at different stages of transition. Obesity is positively associated with socioeconomic factors in Asia but not in Latin America [32]. In South Asia, children from affluent families tend to be heavier than those who are socioeconomically disadvantaged. Changes in lifestyle among children with higher socioeconomic class including unhealthy diets, reduced physical activity and increased sedentary living, reinforced by many of the cultural changes associated with globalization, are the probable underlying causes [48-50]. Children from families having higher socioeconomic class lead a very unhealthy life in this region. They are transported to and from school by car and bus. Sports have been replaced by television, video games and the internet. Parents are busier than ever, household work is done by the servants, families eat fewer home-cooked meals, breakfast skipping is a habit and snacking between meals is the norm. Children from families having lower socioeconomic class do not afford these trends, and tend to be physically active and eat healthy food.

Efforts to stop childhood obesity should be made on all fronts and targeted interventions, designed considering the impact of family environment, should begin early in life [9]. Parents are important in terms of role-modeling physical activity, providing a safe and interesting backyard for children to play in, setting rules about how small screen entertainment is used in the home, guiding behavioral approaches to family food consumption and providing healthy food choices in home [51]. Counseling families on behavior change has been suggested as the best approach to prevent and manage childhood obesity [52]. Prevention and treatment effort following a global approach with proper monitoring and implementation are effective and there is little evidence of negative effects, either physiological or psychological [53-55]. Family-based interventions are routinely recommended for obese school-aged children and school-based programs are recommended for those at risk of being overweight and obese [55]. However, these interventions have been implemented in the developed countries and need to be tested in the resource-poor developing country settings. In Pakistan, public health infrastructure is available to support family- and school-based interventions regarding childhood obesity but these have not been implemented in the country. A National preventive strategy for childhood obesity should be developed and a pilot preventive program should be initiated taking into consideration the impact of family-based factors associated with childhood overweight and obesity. School health and nutrition supervisors working under Pakistan's National maternal, newborn, and child health Program could be involved for implementing school-based initiatives and lady health workers working under Pakistan's National program for family planning and primary health care could be involved for implementing family-based initiatives.

Cross-sectional nature of the study should be considered when interpreting the findings reported and future longitudinal studies are warranted to establish the temporal nature and causality of these associations. The effects of puberty on anthropometric indices were not explored in the present study; however, future studies are suggested in this regard. Although data collection followed a standard protocol, digital scales were not used. Variability in the data ascertainment may have introduced an error into the estimates; however, we do not anticipate large or systematic differences. These findings can be generalized to South Asian primary school children that share the same genetic and environmental factors with the sample.

## Conclusions

Family-based factors were significantly associated with overweight and obesity among school-aged children in Pakistan. Children having higher parental education, both parents working, fewer siblings, less crowded housing and residence in high-income neighborhoods were significantly more likely to be overweight and obese. Higher parental education, living in high-income neighborhoods and fewer siblings were independent predictors of overweight. These findings support the need to design evidence-based child health policy and implement targeted interventions, considering the impact of family-based factors and involving communities.

## Competing interests

The authors declare that they have no competing interests.

## Authors' contributions

MUM, principal investigator, conceived and implemented the study, analyzed and interpreted the data, prepared the manuscript and supervised the entire project. SG, MMS and HMA contributed to the study analysis, interpretation and manuscript preparation. US contributed to the study conception, implementation and analysis. MAS and AMS oversaw the study conception, implementation and manuscript preparation. All authors read and approved the final manuscript.

## Pre-publication history

The pre-publication history for this paper can be accessed here:

http://www.biomedcentral.com/1471-2431/11/114/prepub

## References

[B1] World Health Organization (WHO) Global Strategy on Diet, Physical Activity and Health 2004 Geneva, Switzerland: WHO

[B2] Dietz WH Robinson TN Overweight children and adolescents N Engl J Med 2005 352 2100 2109 10.1056/NEJMcp043052 15901863

[B3] Han JC Lawlor DA Kimm SYS Childhood obesity Lancet 2010 375 1737 1748 10.1016/S0140-6736(10)60171-7 20451244 PMC3073855

[B4] Prentice AM The emerging epidemic of obesity in developing countries Int J Epidemiol 2006 35 93 99 16326822 10.1093/ije/dyi272

[B5] Mushtaq MU Gull S Abdullah HM Shahid U Shad MA Akram J Prevalence and socioeconomic correlates of overweight and obesity among Pakistani primary school children BMC Public Health 2011 11 1 724 10.1186/1471-2458-11-724 21943029 PMC3195095

[B6] Must A Spadano J Coakley EH Field AE Colditz G Dietz WH The disease burden associated with overweight and obesity JAMA 1999 282 1523 9 10.1001/jama.282.16.1523 10546691

[B7] Viner RM Cole TJ Adult socioeconomic, educational, social, and psychological outcomes of childhood obesity: a national birth cohort study BMJ 2005 330 7504 1354 10.1136/bmj.38453.422049.E0 15901644 PMC558281

[B8] World Health Organization (WHO) Obesity: Preventing and Managing the Global Epidemic 2000 Geneva, Switzerland: WHO 11234459

[B9] Reilly JJ Tackling the obesity epidemic: new approaches Arch Dis Child 2006 91 724 6 10.1136/adc.2006.098855 16923855 PMC2082930

[B10] Kleiser C Rosario AS Mensink GBM Prinz-Langenohl R Kurth BM Potential determinants of obesity among children and adolescents in Germany: results from the cross-sectional KiGGS study BMC Public Health 2009 9 46 10.1186/1471-2458-9-46 19187531 PMC2642815

[B11] Salsberry PJ Reagan PB Dynamics of Early Childhood Overweight Pediatrics 2005 116 6 1329 1338 10.1542/peds.2004-2583 16322155 PMC1479091

[B12] Swinburn B Gill T Kumanyika S Obesity prevention: a proposed framework for translating evidence into action Obes Rev 2005 6 23 33 10.1111/j.1467-789X.2005.00184.x 15655036

[B13] Baltrus PT Lynch JW Everson-Rose S Raghunathan TE Kaplan GA Race/Ethnicity, Life-Course Socioeconomic Position, and Body Weight Trajectories Over 34 years: The Alameda County Study Am J Public Health 2005 95 9 1595 1601 10.2105/AJPH.2004.046292 16051936 PMC1449403

[B14] Lamerz A Kuepper-Nybelen J Wehle C Social class, parental education, and obesity prevalence in a study of six-year-old children in Germany Int J Obes (Lond) 2005 29 4 373 80 10.1038/sj.ijo.0802914 15768043

[B15] Danielzik S Czerwinski-Mast M Langnase K Dilba B Muller MJ Parental overweight, socioeconomic status and high birth weight are the major determinants of overweight and obesity in 5-7 y-old children: baseline data of the Kiel Obesity Prevention Study (KOPS) Int J Obes Relat Metab Disord 2004 28 11 1494 502 10.1038/sj.ijo.0802756 15326465

[B16] Kocaoglu B Moschonis G Dimitriou M Parental educational level and cardiovascular disease risk factors in schoolchildren in large urban areas of Turkey: Directions for public health policy BMC Public Health 2005 5 13 10.1186/1471-2458-5-13 15693995 PMC549186

[B17] Huerta M Bibi H Haviv J Scharf S Gdalevich M Parental smoking and education as determinants of overweight in Israeli children Prev Chronic Dis 2006 3 2 A48 16539789 PMC1563963

[B18] Brophy S Cooksey R Gravenor MB Risk factors for childhood obesity at age 5: Analysis of the Millennium Cohort Study BMC Public Health 2009 9 467 10.1186/1471-2458-9-467 20015353 PMC2803190

[B19] Apfelbacher CJ Loerbroks A Cairns J Behrendt H Ring J Krämer U Predictors of overweight and obesity in five to seven-year-old children in Germany: Results from cross-sectional studies BMC Public Health 2008 8 171 10.1186/1471-2458-8-171 18495021 PMC2426699

[B20] Hawkins SS Cole TJ Law C the Millennium Cohort Study Child Health Group An ecological systems approach to examining risk factors for early childhood overweight: findings from the UK Millennium Cohort Study J Epidemiol Community Health 2009 63 147 155 18801795 10.1136/jech.2008.077917 PMC2678539

[B21] Robinson WR Gordon-Larsen P Kaufman JS Suchindran CM Stevens J The female-male disparity in obesity prevalence among black American young adults: contributions of sociodemographic characteristics of the childhood family Am J Clin Nutr 2009 89 1204 12 10.3945/ajcn.2007.25751 19190069 PMC2667464

[B22] Monteiro CA Conde WL Popkin BM Independent effects of income and education on the risk of obesity in the Brazilian adult population J Nutr 2001 131 881S 886S 11238779 10.1093/jn/131.3.881S

[B23] Kries R Bolte G Baghi L Toschke AM GME Study Group Parental smoking and childhood obesity--is maternal smoking in pregnancy the critical exposure? Int J Epidemiol 2008 37 210 216 18056122 10.1093/ije/dym239

[B24] Hakeem R Socio-economic differences in height and body mass index of children and adults living in urban areas of Karachi, Pakistan Eur J Clin Nutr 2001 55 400 406 10.1038/sj.ejcn.1601172 11378815

[B25] Sidhu S Marwah G Prabhjot Prevalence of overweight and obesity among the affluent adolescent school children of Amritsar, Punjab Coll Antropol 2005 29 53 55 16117299

[B26] Chhatwal J Verma M Riar SK Obesity among pre-adolescent and adolescents of a developing country (India) Asia Pac J Clin Nutr 2004 13 231 235 15331333

[B27] Wang Y Cross-national comparison of childhood obesity: the epidemic and the relationship between obesity and socioeconomic status Int J Epidemiol 2001 30 1129 1136 10.1093/ije/30.5.1129 11689534

[B28] Veugelers PJ Fitzgerald AL Prevalence of and risk factors for childhood overweight and obesity CMAJ 2005 173 6 607 613 10.1503/cmaj.050445 16157724 PMC1197160

[B29] Melnick TA Rhoades SJ Wales KR Cowell C Wolfe WS Overweight school children in New York City: prevalence estimates and characteristics Int J Obes 1998 22;7 13 10.1038/sj.ijo.0800537 9481594

[B30] Van Lenthe FJ Mackenbach JP Neighbourhood deprivation and overweight: the GLOBE study Int J Obes Relat Metab Disord 2002 26 234 40 10.1038/sj.ijo.0801841 11850756

[B31] Willms JD Tremblay MS Katzmarzyk PT Geographic and demographic variation in the prevalence of overweight Canadian children Obes Res 2003 11 668 73 10.1038/oby.2003.95 12740457

[B32] INCLEN Body mass index and cardiovascular disease risk factors in seven Asian and five Latin American centers data from the International Clinical Epidemiology Network (INCLEN) Obes Res 1996 4 221 228 8732956 10.1002/j.1550-8528.1996.tb00540.x

[B33] Thorpe LE List DG Marx T May L Helgerson SD Frieden TR Childhood obesity in New York City elementary school students Am J Public Health 2004 94 9 1496 500 10.2105/AJPH.94.9.1496 15333301 PMC1448480

[B34] Kelishadi R Ardalan G Gheiratmand R Association of physical activity and dietary behaviours in relation to the body mass index in a national sample of Iranian children and adolescents: CASPIAN Study Bull World Health Organ 2007 85 19 26 10.2471/BLT.06.030783 17242754 PMC2636217

[B35] Whelton H Harrington J Crowley E Kelleher V Cronin M Perry IJ Prevalence of overweight and obesity on the island of Ireland: results from the North South Survey of Children's Height, Weight and Body Mass Index, 2002 BMC Public Health 2007 7 187 10.1186/1471-2458-7-187 17672893 PMC1950090

[B36] ZT Mechanical Physician Scale, East High Scales, China Scale Manufacturer, Nanjing, China http://www.easthighscale.com/ZT-Mechanical-Physician-Scale.html

[B37] Sweeting H Measurement and definitions of obesity in childhood and adolescence: a field guide for the uninitiated Nutr J 2007 6 32 10.1186/1475-2891-6-32 17963490 PMC2164947

[B38] Durkin MS Islam S Hasan ZM Zaman SS Measures of socioeconomic status for child health research: comparative results from Bangladesh and Pakistan Soc Sci Med 1994 38 1289 1297 10.1016/0277-9536(94)90192-9 8016692

[B39] Galobardes B Shaw M Lawlor DA Lynch JW Smith GD Indicators of socioeconomic position (part 2) J Epidemiol Community Health 2006 60 2 95 101 10.1136/jech.2004.028092 16415256 PMC2566160

[B40] World Health Organization WHO Child Growth Standards http://www.who.int/growthref/en/

[B41] Butte NF Garza C de Onis M Evaluation of the feasibility of international growth standards for school-aged children and adolescents J Nutr 2007 137 153 157 17182818 10.1093/jn/137.1.153

[B42] Shrewsbury V Wardle J Socioeconomic Status and Adiposity in Childhood: A Systematic Review of Cross-sectional Studies 1990-2005 Obesity (Silver Spring) 2008 16 275 284 10.1038/oby.2007.35 18239633

[B43] Ukwuani F Suchindran C Implications of women's work for child nutritional status in sub-Saharan Africa: A case study of Nigeria Soc Sci Med 2003 56 10 2109 2121 10.1016/S0277-9536(02)00205-8 12697201

[B44] Monteiro CA Conde WL Lu B Popkin BM Obesity and inequities in health in the developing world Int J Obes 2004 28 1181 1186 10.1038/sj.ijo.0802716 15211362

[B45] Wang Y Beydoun MA The Obesity Epidemic in the United States--Gender, Age, Socioeconomic, Racial/Ethnic, and Geographic Characteristics: A Systematic Review and Meta-Regression Analysis Epidemiol Rev 2007 29 6 28 10.1093/epirev/mxm007 17510091

[B46] O'Dea JA Gender, ethnicity, culture and social class influences on childhood obesity among Australian schoolchildren: implications for treatment, prevention and community education Health Soc Care Community 2008 16 3 282 290 10.1111/j.1365-2524.2008.00768.x 18328051

[B47] Miech RA Kumanyika SK Stettler N Link BG Phelan JC Chang VW Trends in the association of poverty with overweight among US adolescents, 1971-2004 JAMA 2006 295 2385 2393 10.1001/jama.295.20.2385 16720824

[B48] Wang Y Lobstein T Worldwide trends in childhood overweight and obesity Int J Pediatr Obes 2006 1 11 25 10.1080/17477160600586747 17902211

[B49] Chopra M Galbraith S Darnton-Hill I A global response to a global problem: the epidemic of overnutrition Bull World Health Organ 2002 80 952 958 12571723 PMC2567699

[B50] Popkin BM An overview on the nutrition transition and its health implications: the Bellagio meeting Public Health Nutr 2002 5 93 103 12027297 10.1079/phn2001280

[B51] Spurrier NJ Margarey AA Golley R Curnow F Sawyer MG Relationships between the home environment and physical activity and dietary patterns of preschool children: a cross-sectional study Int J Behav Nutr Phys Act 2008 5 31 10.1186/1479-5868-5-31 18513416 PMC2432071

[B52] Plourde G Preventing and managing pediatric obesity Can Fam Physician 2006 52 3 322 328 16572577 PMC1479709

[B53] American Dietetic Association Ritchie LD Crawford PB Hoelscher D Sothern MS Position of the American Dietetic Association: individual-, family-, school-, and community-based interventions for pediatric overweight J Am Diet Assoc 2006 106 925 945 16812927 10.1016/j.jada.2006.03.001

[B54] Epstein LH Myers MD Raynor H Saelens BE Treatment of pediatric obesity Pediatrics 1998 101 3 554 570 12224662

[B55] Summerbell CD Waters E Edmunds LD Kelly S Brown T Campbell KJ Interventions for preventing obesity in children Cochrane Database of Systematic Reviews 2005 3 CD001871 10.1002/14651858.CD001871.pub2 16034868

